# Selecting the right therapeutic target for kidney disease

**DOI:** 10.3389/fphar.2022.971065

**Published:** 2022-11-02

**Authors:** Lisa Buvall, Robert I. Menzies, Julie Williams, Kevin J. Woollard, Chanchal Kumar, Anna B. Granqvist, Maria Fritsch, Denis Feliers, Anna Reznichenko, Davide Gianni, Slavé Petrovski, Claus Bendtsen, Mohammad Bohlooly-Y, Carolina Haefliger, Regina Fritsche Danielson, Pernille B. L. Hansen

**Affiliations:** ^1^ Bioscience Renal, Research and Early Development, Cardiovascular, Renal and Metabolism, BioPharmaceuticals R&D, AstraZeneca, Gothenburg, Sweden; ^2^ Bioscience Renal, Research and Early Development, Cardiovascular, Renal and Metabolism, BioPharmaceuticals R&D, AstraZeneca, Cambridge, United Kingdom; ^3^ Translational Science and Experimental Medicine, Research and Early Development, Cardiovascular, Renal and Metabolism, BioPharmaceuticals R&D, AstraZeneca, Gothenburg, Sweden; ^4^ Functional Genomics, Discovery Sciences, R&D, AstraZeneca, Cambridge, United Kingdom; ^5^ Centre for Genomics Research, Discovery Sciences, R&D, AstraZeneca, Cambridge, United Kingdom; ^6^ Data Sciences & Quantitative Biology, Discovery Sciences, R&D, AstraZeneca, Cambridge, United Kingdom; ^7^ Translational Genomics, Discovery Sciences, R&D, AstraZeneca, Gothenburg, Sweden

**Keywords:** chronic kidney disease, validation, systems biology, omics, machine learning, drug discovery, artificial intelligence

## Abstract

Kidney disease is a complex disease with several different etiologies and underlying associated pathophysiology. This is reflected by the lack of effective treatment therapies in chronic kidney disease (CKD) that stop disease progression. However, novel strategies, recent scientific breakthroughs, and technological advances have revealed new possibilities for finding novel disease drivers in CKD. This review describes some of the latest advances in the field and brings them together in a more holistic framework as applied to identification and validation of disease drivers in CKD. It uses high-resolution ‘patient-centric’ omics data sets, advanced *in silico* tools (systems biology, connectivity mapping, and machine learning) and ‘state-of-the-art‘ experimental systems (complex 3D systems *in vitro*, CRISPR gene editing, and various model biological systems *in vivo*). Application of such a framework is expected to increase the likelihood of successful identification of novel drug candidates based on strong human target validation and a better scientific understanding of underlying mechanisms.

## 1 Introduction

Chronic kidney disease (CKD) is an umbrella term for a variety of renal diseases with different etiologies, usually diagnosed on the basis of clinical and/or histopathological features, but commonly associated with longstanding diabetes and hypertension (Kidney Disease: Improving Global Outcomes, 2013). CKD is divided into five stages, 1–5, according to the estimated glomerular filtration rate (eGFR), as a measure of overall renal function, and degree of any associated albuminuria, which both correlate with increasing mortality risk (Webster et al., 2017). However, the current clinical and histological features of the different renal diseases that make up CKD do not relate to a particular pathway or factor that can define the underlying disease mechanism(s), and thereby determine a specific therapeutic intervention. This gap between the clinical and histolological classification of renal injury and the underlying drivers of disease makes use of the current CKD classification alone problematic for drug discovery (Hall and Himmelfarb, 2017). Furthermore, the structural and functional complexity of the kidney, which comprises a variety of cell types - glomerular, tubular, interstitial, and vascular—highlights the need for a deeper understanding of the underlying biology and physiology, and requirement for a broad array of tools for target validation.

Drug discovery in CKD has proved challenging. The global burden of CKD is increasing worldwide with a country-by-country prevalence ranging from 5% to 14%; CKD is ranked the fourth in a list of growing mortalities in 2020 (The Lancet Kidney Campaign, 2020). Moreover, 7.6% of all deaths from cardiovascular disease (CVD) can be attributed to CKD (G. B. D. Chronic Kidney Disease Collaboration, 2020). Despite recent progress in the treatment of CKD, such as the approval of sodium-glucose cotransporter-2 (SGLT2) inhibitors (Bakris et al., 2020; Heerspink et al., 2020), major therapeutic advances are still required to halt and reverse CKD.

The molecular drivers of renal disease are diverse and may involve over 100 biological pathways (Martini et al., 2014) and this complexity makes it difficult to recapitulate disease pathways experimentally. Two-dimensional culture of particular renal cell types has advanced our understanding of renal biology, but many components are lacking, such as cell-to-cell contact, cell-matrix interactions, and the effects of flow or pressure. Moreover, with at least 25 different cell types (Park et al., 2019), a faithful simulation of the kidney *ex vivo* is unfeasible at present. Therefore, efforts so far have focused on building particular capabilities that allow us to model different aspects of renal pathophysiology and anatomy in disease (Balzer et al., 2022), including omics-defined translatable primary human renal cells *in vitro* and better genomic characterization of animal models *in vivo*.

The imprecision of the current CKD classification, the complexity of the underlying renal pathophysiology, and lack of adequate translatability from preclinical findings to clinical readouts are the major challenges when identifying relevant disease drivers. Recent technological innovations and the application of multiple target identification approaches are opening up new avenues for researchers in the quest to identify and validate the right targets for CKD. Expanding collections of omics data, together with human efficacy trials are invaluable resources for ‘back-translation’ in early target identification (King et al., 2019). Advances in artificial intelligence (AI) and systems biology provide the opportunity to develop machine-learning algorithms to combine diverse sources of patient data for a more patient-centric and holistic analysis that can be applied to target identification. In addition, many studies have shown that when a target can be linked to genetic evidence in disease, or a firm understanding of the role of a target in the etiology of a disease, these are less likely to fail due to insufficient efficacy (Kamb et al., 2013; Plenge et al., 2013; Cook et al., 2014; Hurle et al., 2016; King et al., 2019). In parallel with ‘big data’ discovery, efficient mechanistic target validation requires information from a broad range of sources, including *in silico*, *in vitro,* and *in vivo* research. Bioinformatic analysis applied to available human CKD data and combined with readouts from complex 3D models *in vitro* capturing cellular cross-talk and placed in a framework based on transcriptomic readouts from the why rat animal model *in vivo* are now becoming essential for renal drug discovery.

**FIGURE 1:**
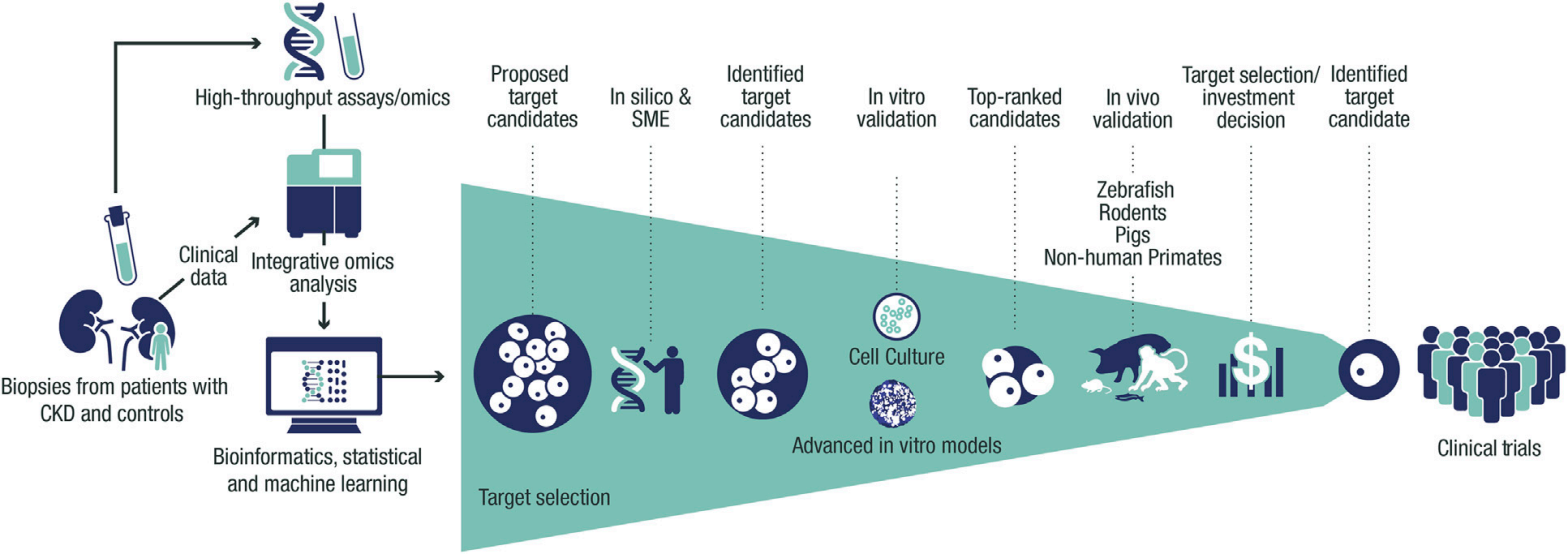
Framework for target identification and validation in CKD. Potential CKD targets identified from human data *via in silico* and SME approaches are prioritized using thorough validation in *vitro* and *in vivo* systems, facilitating target selection. Target identification starts with the collection of biopsies, urine, and blood from patients with CKD and controls from clinical trials and collaborations. Omics data (genetic, transcriptomic, proteomic, and metabolomic) are then generated from these samples and processed with integrative omics analyses followed by bioinformatic and statistical analyses and machine learning. This data processing results in a list of CKD targets that is assessed *in silico* to build further evidence of human target–disease associations. The shortlisted targets are then prioritized by applying biologically relevant *in vitro* validation in cultured cells and advanced *in vitro* models. The targets with the strongest supportive data are then validated further *in vivo* to build proof-of-mechanism and proof-of-principle in CKD before being presented for target selection and investment decision to enter the portfolio. CKD, chronic kidney disease; SME, subject-matter expert.

The challenging nature to identify disease drivers in CKD demands a contemporary holistic approach (Figure 1). This framework is based on human target validation, starting with the generation of a list of potential targets that is derived from patient-relevant data for hypothesis testing, followed by validation using various experimental platforms. Positive validation readouts from multiple testing can facilitate data-driven selection of candidate CKD targets. In this article, we describe how combining existing knowledge of renal diseases with recent technological and scientific advances can help to address the challenges we face in identifying the optimal drug targets for treating CKD.

## 2 Identifying new disease drivers through patient-derived big data

The highly heterogeneous and progressive nature of CKD makes it amenable to using big data from large human populations to transform the way we identify potential disease drivers, better understand disease progression, and ultimately increase the success rate in drug development for this growing unmet medical need. Retrospective analyses based on data from human genetic studies, such as familial studies and genome-wide association studies (GWAS), have shown that drugs with targets supported by human genetics have an above-average chance of clinical success (Kamb et al., 2013). In CKD, the identification of rare variants, using collapsing analyses of exome sequences, can validate known disease-causing genes and identify candidate genes and modifiers (Cameron-Christie et al., 2019). Therefore, human genetics can play a key role in: i) identifying drug targets with strong human target validation for therapeutic intervention and impact, ii) validating the mechanisms of action of existing drug candidates to reduce their risk of failing, iii) implementing precision medicine strategies to identify patients with molecular diagnoses that are more likely to benefit from targeted therapies, and iv) detecting potential prohibitive drug interactions and adverse effects.

Beyond the foundation of genomics, other patient-derived large data sets that include transcriptomics, proteomics, and metabolomics, and which contain more temporal and tissue-spatial information, are critical to deciphering the downstream complexity of disease defined by the interplay between genetics and the environment. Various molecular data sets can be generated from clinical trial participants to build an understanding from gene to protein, and the metabolomic signature in patients with CKD (Eddy et al., 2020).

One of the greatest enablers for big data and multi-omic collections is increased collaboration. One example is the UK Biobank in which many pharmaceutical companies have joined a pre-competitive consortium to generate exome and genome sequence data in approximately 500,000 participants, an unparalleled clinical and genomic resource (Bycroft et al., 2018). CKD-specific collaborations have also been founded, including the Renal Pre-competitive Consortium (RPC^2^), in which data, resources, and expertise in molecular target identification are shared across academia and the pharmaceutical industry with the aim of accelerating novel drug development for CKD through a systems biology approach (Tomilo et al., 2018). This consortium-generated pre-competitive material (data sets and unbiased analyses) is shared equally among all partners and then with the wider scientific community, while the industry partners can conduct internal competitive research for the development of targets at their own discretion and that is fully amenable to intellectual property protection. This model has been followed by the establishment in recent years of several well-curated national CKD cohort studies for long-term patient follow-up and the collection of longitudinal clinical data and biosamples of blood and urine, and in some cases also renal tissue samples (Tomilo et al., 2018).

## 3 Bioinformatics-based prioritization of disease drivers

**FIGURE 2:**
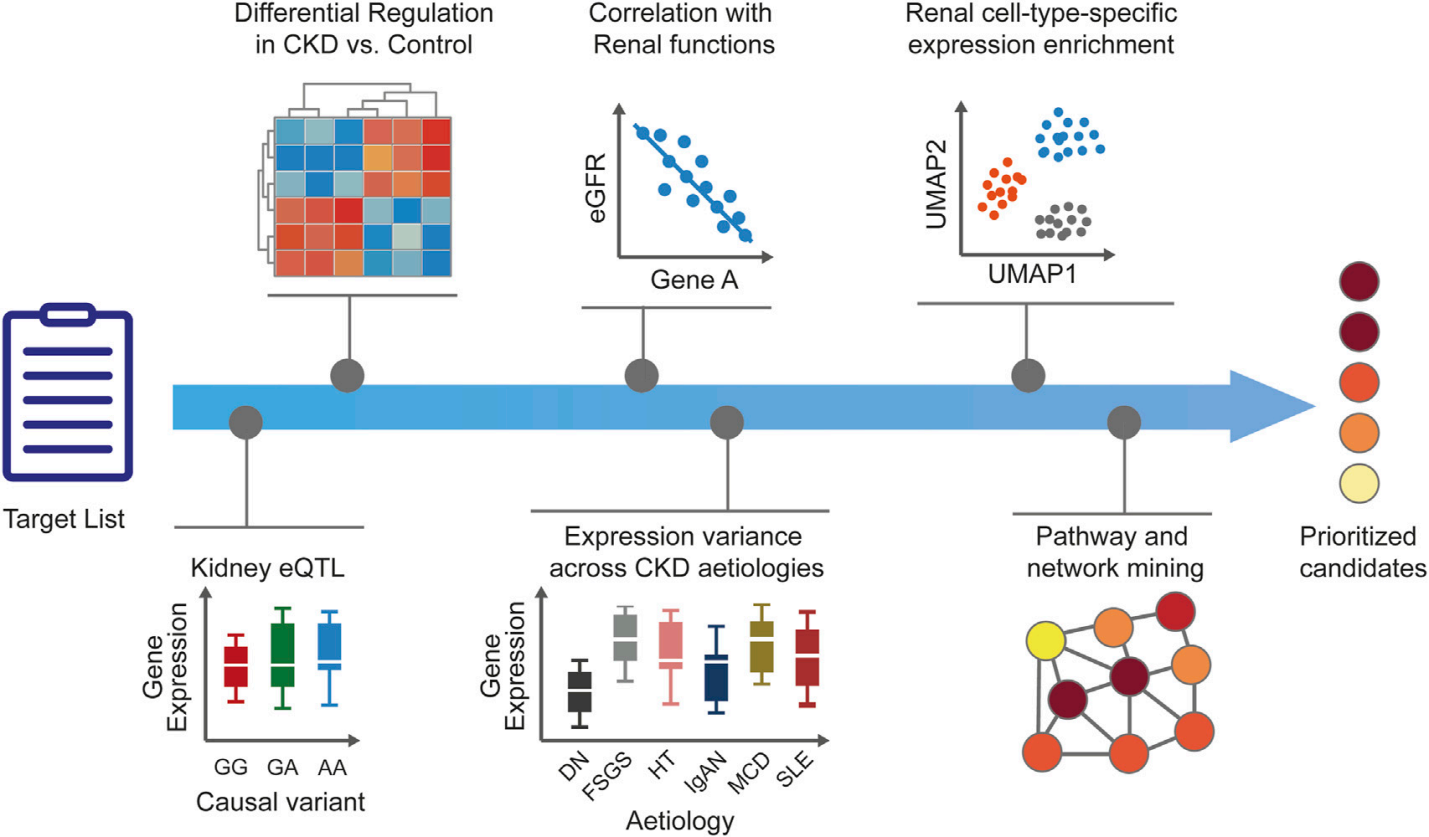
Human target validation and prioritization. After generation of CKD target lists, additional disease-relevant evidence is added to generate testable hypotheses and to prioritize the candidates. Tissue-specific expression enrichment and expression modulation in disease *versus* healthy states help to ascertain the role of targets in CKD. Correlation of targets with renal functional biomarkers and parameters is ascertained and target expression across CKD stages or etiologies is explored to add confidence around disease relevance. Prediction of target kidney cell type is useful for guiding downstream *in vitro* validation and assay selection. Pathway and network analyses can provide additional biological context for dysregulated cellular mechanisms and help infer potential mechanisms of action. The accumulated evidence supporting human target validation and mechanism of action results in a set of prioritized candidates for further experimental validation. CKD, chronic kidney disease; DN, diabetic nephropathy; eGFR, estimated glomerular filtration rate; eQTL, expression quantitative trait loci; FSGS, focal segmental glomerulosclerosis; HT, hypertensive nephropathy; IgA, immunoglobulin A nephropathy; MCD, minimal change disease; MGN, membranous glomerulonephritis; RPGN, rapid progressive glomerulonephritis; SLE, systemic lupus erythematosus.

Bioinformatic approaches are used extensively to gather CKD supporting evidence for targets, including their importance as drivers of CKD. Bioinformatics continues to play a key role in the success of omics and has integrated itself seamlessly into the fabric of contemporary data-driven biology. The ability to generate multidimensional omic data sets from genomic, blood, urine, and kidney tissue sample analyses of patients with CKD has opened up new possibilities for data-driven hypothesis generation (Saez-Rodriguez et al., 2019). Various systematic knowledge-mining approaches and comprehensive functional analysis of patient-derived omic and clinical data sets can now be employed to facilitate CKD target identification, validation, and prioritization (Figure 2).

Analysis of omic data is a multifaceted endeavor that integrates many aspects of bioinformatics, statistics, and machine learning (Hastie et al., 2009). Algorithms and workflows are employed to conduct specific aspects of data analysis, including data normalization (Kohl et al., 2014; Abbas-Aghababazadeh et al., 2018), exploratory multivariable data analysis (Gehlenborg et al., 2010), and systems-level omics integration (Mitra et al., 2013; Karczewski and Snyder, 2018). Platform-dependent omics readouts (transcriptomics, proteomics, and metabolomics) necessitate specialized data normalization to avoid technical biases obscuring biological patterns (Tyanova et al., 2016; Yamada et al., 2020), and eventually produce a data matrix that typically represents the abundance values for molecular entities (genes, proteins, and metabolites) across biological samples. Once normalized, unbiased, hypothesis-free data exploration usually begins by using various unsupervised dimensionality reduction methods (Bartenhagen et al., 2010) that permit visualization of large multidimensional data sets by summarizing thousands of variables in a few principal components that can be plotted in 2D space. Even at this initial stage, underlying patterns can be identified within the data structure by revealing the grouping of samples or their molecular features, and sometimes aided by use of hierarchical clustering.

Case-control design remains a mainstay of observational cohort studies in which omics data are generated from patients with CKD (broad or specific disease characteristics) and controls (healthy individuals or patients with non-renal disease) with the aim of making comparisons. In such a setting, differential abundance/expression analyses are used to uncover molecular features that are modulated in CKD *versus* control samples. Directionality of differences and changes—up- or down-regulation—sheds light on the activity state of biological processes, as well as guiding the subsequent therapeutic approach, namely development of an antagonist or agonist compound, respectively, and choosing the most appropriate modality.

Hypothesis-free data analysis methods typically yield long lists of disease-associated molecular features (such as genes), which can be ranked by, for example, statistical significance or magnitude of effect, but cannot easily be tested experimentally or have unknown biological meaning. Therefore, a substantial part of data analytics is devoted to the prioritization of candidate features to shortlist the most promising potential drug targets for subsequent validation *in vitro* and *in vivo* (Moreau and Tranchevent, 2012; Vitsios and Petrovski, 2020). The prioritization is based on annotation of those candidates with sufficient disease-related evidence to support generation of a testable hypothesis. Patient clinical characteristics are key to the interpretation of omics patterns because hypothesis-based analyses depend on a biological understanding of the disease and include testing of candidate targets for their correlations with renal function (serum creatinine, cystatin C, glomerular filtration rate [GFR], albuminuria, or proteinuria), disease progression rate, CKD stage, and histopathological diagnosis. When follow-up clinical data are available, longitudinal analyses for association with changes in renal function over time (GFR slope) and prediction of later outcomes (worsening proteinuria, cardiovascular morbidity, onset of renal failure, and mortality) are valuable in understanding the relative importance of a chosen target.

For selected candidate targets, pathway analyses and network biology approaches are used to provide additional biological context for dysregulated cellular mechanisms and to infer potential mechanisms of action (Gehlenborg et al., 2010; Choobdar et al., 2019; Reimand et al., 2019). Further hypothesis-driven analyses can inform subsequent steps for preclinical target validation. For example, prediction of the target cell type is useful in guiding the choice of cellular model *in vitro*, kidney enrichment of intra-renal targets can inform pharmacokinetic aspects and safety issues, and the presence of orthologous genes, pathway conservation, and consistent directionality modulation in disease models can support human-to-animal translatability and selection of animal models *in vivo*. Prioritized candidate targets can then enter the pipeline of preclinical testing for further target validation.

## 4 *In vitro* target validation

The appropriate use of translatable models *in vitro* and tools for preclinical target validation can build confidence in novel CKD targets. Preclinical target validation can include building an understanding of the mechanism of action of a particular target and an understanding of its molecular network. Confidence in a candidate target identified *in silico* can be gained by demonstrating pathway activity in human cell-based renal disease models *in vitro*.

As mentioned earlier, the complexity of kidney disease, which may involve the dysfunction of several different cell types in the glomerular, tubulo-interstitial, and vascular compartments, makes it challenging to identify translatable models in which to define the mechanism of action of potential targets in CKD. To facilitate selection of the best validation system, human omic data sources may be analyzed in combination with transcriptomic, proteomic, and metabolomic data *in vitro*, both in cell culture systems and in more advanced cellular models. In this way, we can identify the appropriate cell type, model system, and the right renal stressor for the target pathway, in the same way as big data and bioinformatic analyses can identify these elements in patients with CKD. CRISPR screening in renal cells can help to handle the increasing number of targets arising from big data, making it easier to triage multiple targets and select the most promising candidate for further validation.

A renal toolbox must try to mirror the renal multiplicity of cell types, the complexity of their interactions, cross-talk with other organ systems, including effects on metabolism, the microbiome, and immune system. Recently, significant progress has been made to improve the translatability of *in vitro* renal systems, such as the generation of podocytes derived from human induced pluripotent stem cells (iPSCs) that have gene expression signatures resembling adult human podocytes more closely than available podocyte cell lines (Yoshimura et al., 2019). In addition, primary and immortalized cell lines (Wieser et al., 2008) of several different glomerular, tubular, vasculature, and immune cell types may be used in validation assays. Co-culture systems are also advancing for studying the interplay among renal cell types and with other cells such as immune cells.

**FIGURE 3:**
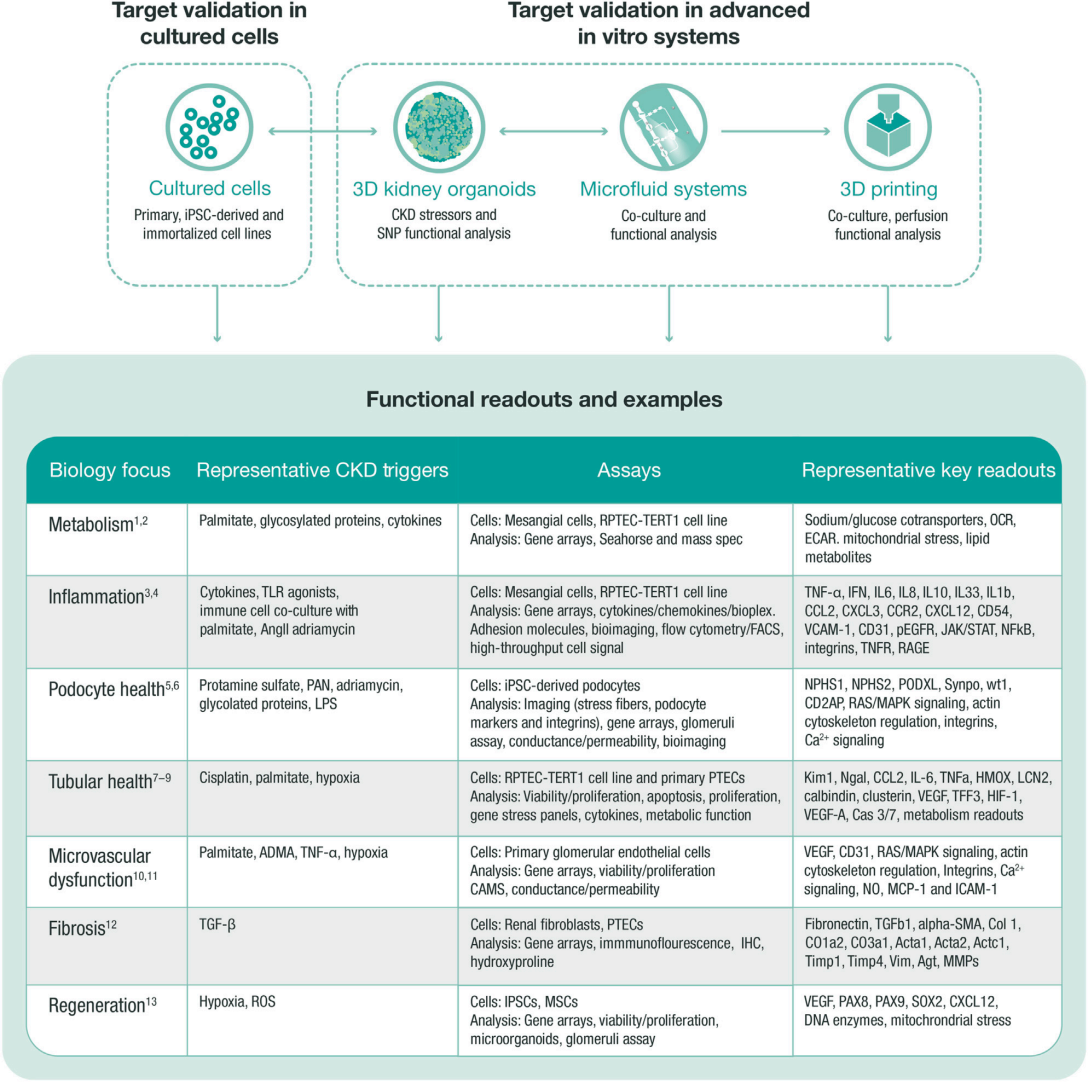
*In vitro* target validation process. *In vitro* target validation in cultured cells is used as a first approach to validate and screen several targets, aiming to triage targets with strong support for further target validation in advanced *in vitro* systems. Data from all systems feed into each other to select the most translatable model and define the correct stressor that regulates target and pathway. An *in vitro* target validation toolbox may comprise various assays, stressors, and readouts, which are chosen based on the disease biology of the target. ADMA, asymmetric dimethylarginine (arginine metabolite); CKD, chronic kidney disease; ECAR, extracellular acidification rate; HGEC, human glomerular endothelial cell; HMOX, hem oxygenase; IHC, immunohistochemistry; iPSC, induced pluripotent stem cell; MMPs, matrix metalloproteinases; MSC, mesenchymal stem cell; OCR, oxygen consumption rate; PAN, pyromycin aminonucleoside; PTEC, proximal tubular epithelial cell; RPTEC, renal proximal tubular epithelial cell; SNP, single nucleotide polymorphism; TGF, transforming growth factor; TLR, toll-like receptor; ROS, reactive oxygen species. 1. Faivre A et al. Front Med (Lausanne) 2021; 8:742072; 2. Imasawa T et al. The International Journal of Biochemistry and Cell Biology 2013; 45:2109–2118.3. Oates JC et al. American Journal of Physiology-Renal Physiology 2022; 322:F309-F321.4. Tang SCW et al. Nature Reviews Nephrology 2020; 16:206–222.5. Lee HW et al. Journal of the American Society of Nephrology 2015; 26:2741–2752.6. Perico L et al. Nature Reviews Nephrology 2016; 12:692–710.7. Prozialeck WC et al. Pharmacology and Therapeutics 2007; 114:74–93.8. Slyne J et al. Nephrology Dialysis Transplantation 2015; 30:iv60-iv67.9. Wieser M et al. American Journal of Physiology-Renal Physiology 2008; 295:F1365-F1375.10. Jourde-Chiche N et al. Nature Reviews Nephrology 2019; 15:87–108.11. Sol M et al. Front Pharmacol 2020; 11:573557.12. Liu Y. Kidney International 2006; 69:213–217.13. Yun CW et al. International Journal of Molecular Sciences 2019; 20:1619.

A stepwise approach may be implemented to guide the experimental design for validation of renal targets *in vitro* (Box 1). This process facilitates selection of the most appropriate cell models, renal stressors, and CKD readouts to define mechanism(s) of action, as well as the affected renal compartment to optimize translatability (Figure 3). By using an advanced and translatable *in vitro* toolbox, clear assay design, and appropriate readouts, renal validation *in vitro* can narrow down a list of targets and help select number of promising candidates for further validation in advanced *in vitro*, *ex vivo*, and *in vivo* systems.

**FIGURE 4:**
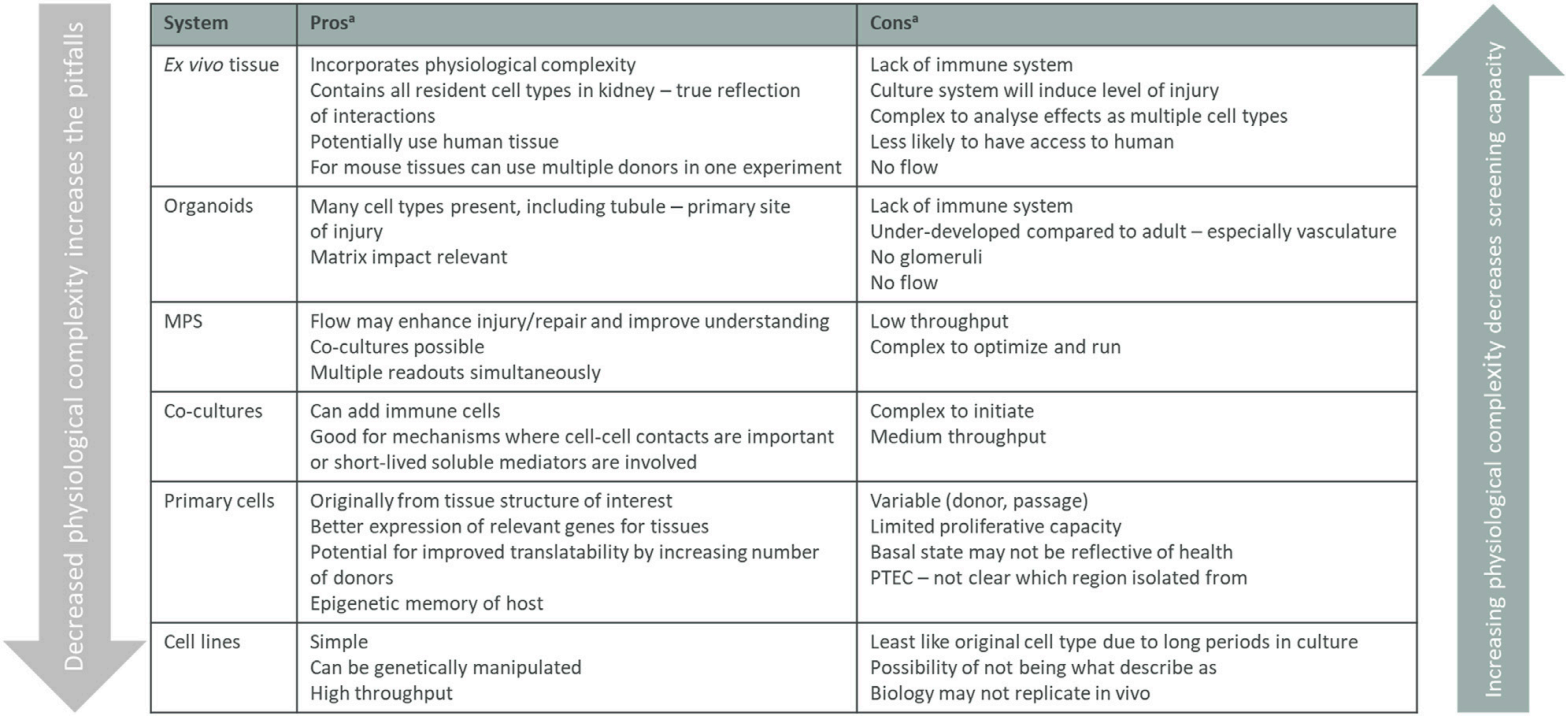
Summary of *in vitro* validation models. Listed *in vitro* models arranged from the assay containing the most tissue complexity down to single cells, listing the pros and cons for the different models. The physiological complexity of the different models impacts both the screening capacity of the assay and what biology that translates. MPS, micro-physiological systems. ^a^considerations are listed in a step-wise manner and main considerations only are stated.

All model systems have their limitations and for the different cell culture models you need to be aware that targets, pathways, and functions can be changed or even lost when cells are removed from their natural environment. The interactions with neighboring cells, changed physiological conditions, and loss of 3D structure can affect the readouts from a simplified 2D culture system and have, for example, been shown to impact gene regulation in proximal tubular cells. The 2D models *in vitro* should therefore not stand alone in a target validation package but be combined with other models described below. 2D models are useful systems before setting up more complex, low throughput, 3D systems *in vitro* system, and models *in vivo*. Each system can play a role in adding to knowledge of mechanisms and the pros and cons are all summarized in Figure 4 and how and when they are used are based on the biology question being asked.

### 4.1 Functional genomics

The discovery of CRISPR technology and its applications to large-scale genetic loss-of-function screens, also known as functional genomics, holds great promise for the identification of novel validated drug targets, including in CKD. Functional genomics screens allow the systematic perturbation of large numbers of genes or proteins, revealing cellular phenotypes that allow for an inference of gene function. The success of functional genomic platforms in driving the identification of the most relevant targets in renal disease will be determined by advances based on three strategic pillars that are the foundation of functional genomics: (i) development of more advanced *in vitro* systems, (ii) creation of validated screening libraries and technologies to alter gene and protein function, and (iii) establishment of ‘end-to-end’ computational pipelines that can facilitate the quantitative analysis of cellular phenotypes resulting from genetic or other perturbations.

According to these three strategic pillars the choice and development of the appropriate *in vitro* system will be critical for the identification of translatable targets in CKD. The value generated by functional genomic efforts will be directly proportional to the translatability of the cellular models employed in screening campaigns in which the targets were originally discovered. Consequently, there is a strong drive among researchers in the field to use cellular systems *in vitro* that can closely recapitulate disease-relevant phenotypes, rather than use easier to screen 2D cell lines with limited their physiological relevance. A successful example is the use of CRISPR methodology with iPSC-derived podocytes and primary renal cells, such as implementation of the ObLiGaRe doxycycline inducible (ODIn) Cas9 system in iPSCs (Lundin et al., 2020).

## 5 Advanced *in vitro* systems

Advanced *in vitro* systems aim to bridge the gap between *in vitro* and *in vivo* by facilitating the exploration of mechanisms that rely on factors such as cross-talk between cells, cell-matrix interactions or changes in pressure or flow that are not present in 2D cultures. The improved maturity of cells in these advanced systems means that basal expression levels of many genes are reduced, providing a cleaner background on which to detect pathophysiological changes in gene expression. *In vivo*, the presence of multiple cell types can either protect the target cell from injury or cascade damage throughout its structure. These nuances in advanced models allow us to answer some intricate questions without the use of *in vivo* models, thereby achieving a reduction in the use of experimental animals. In addition, the same cellular manipulations applied to 2D systems can still be used.

The toolbox available for renal drug discovery is growing with the advent of microphysiological systems that include both glomerular and tubular structures, as well as 3D bio-printed tubules, and more complex renal organoids. From these systems investigation of the target choice can progress to *ex vivo* models, including isolated glomeruli, blood vessels, and tubules, through to kidney slices. Each model has its own advantages and increases our understanding of a target’s impact on disease biology—knowledge that would not be gained from simpler systems.

### 5.1 Glomerular and tubular surrogates

Several different approaches have been taken to tackle the problem of mimicking glomerular structure and function, including co-culture membrane-based chips (Zhou et al., 2016; Musah et al., 2017), in-gel self-aggregation methods (Waters et al., 2017), and isolated glomeruli on chips (Wang et al., 2017). The addition of flow and a more physiological matrix has given rise to structures that show a greater resemblance to their intact homolog (Musah et al., 2017). The replacement of thick artificial membranes with structures having a more natural morphology has also improved functionality (Slater et al., 2011). The main readouts from such systems are imaging, transcriptomics, and permeability. A further step forward has been allowing the cells to generate their own structures (Waters et al., 2017), which can give rise to tubular structures surrounded by a supporting matrix of cells and interstitium; however, the ability to perfuse these structures is so far limited. Disease phenotypes have been modeled in many of these systems, including diabetic (Wang et al., 2017), hypertensive (Zhou et al., 2016) and fibrotic kidney disease (Waters et al., 2017). However, while these are of interest for drug development, they frequently lack complexity or appropriate controls, such as adjusting for the osmotic stress of high glucose. Despite these limitations, interesting findings can be made from co-culture systems, such as the ability of podocytes to reduce endothelial inflammation (Kuravi et al., 2014).

The renal tubule may seem to be a less difficult structure to replicate than the glomerulus. However, it too comprises several different cell types with segment-specific properties, mainly to do with fluid, electrolyte and solute transport, and is surrounded by the ‘black box’ of the interstitium, which is ill-defined and even harder to reproduce. Therefore, a reductionist approach has been taken and the majority of systems have chosen to model the proximal tubule. These range from monocultures on membranes under flow (Jang et al., 2013) to more complex 3D bio-printed tubules with associated vascular structures (Homan et al., 2016). Characterization has highlighted the difficulties of fully replicating the *in vivo* characteristics of tubular cells and with no system to date expressing the full gamut of receptors/transporters or with their correct localization. Introducing flow has certainly improved this, as has the addition of matrix, as well as endothelial cells, which all improve the phenotype of tubular epithelial cells (Aydin et al., 2008; Miya et al., 2011). However, the majority of these model tubular systems have been used to screen for drug nephrotoxicity and changes in drug metabolism and secretion (DesRochers et al., 2013; Soo et al., 2018). Disease model development has not been pursued so far; however, the system can replicate epithelial–mesenchymal transition (Zhou et al., 2014).

### 5.2 Organoids

Pluripotent stem cells can be differentiated to form 3D kidney organoids representative of a first trimester developing fetal kidney. These complex self-aggregating structures usually contain at least 11 different cell types (Harder et al., 2019), representing the major nephron segments, including cells of the glomerulus (podocytes), tubule (proximal, distal, and connecting segments), vasculature (although often scant), and interstitial stroma. 3D bioprinting technology offers the opportunity to improve morphology and throughput (Higgins et al., 2018). The addition of flow has been shown to improve vascularization (Homan et al., 2019).

Kidney organoids are a robust model in which to study human kidney development (Little et al., 2016), a potential source of human kidney cells for bioengineering and regeneration (personalized medicine), a system for toxicological assessment of pharmaceuticals (Czerniecki et al., 2018), and a tool for drug development. Numerous readouts can be used in these systems. CRISPR-Cas9 gene editing in iPSCs has produced several fluorescence reporter cell lines that allow easy visualization and lineage tracking of individual cell populations (Borestrom et al., 2018), as well as the inducible ODIn Cas9 system (Lundin et al., 2020) for speedy target validation in iPSC-derived organoids. Furthermore, high-throughput screening platforms and automated multidimensional phenotyping analysis permit measurement of multiple parameters with multiple stimuli (Ramm et al., 2016). Organoids can also be used to model human renal diseases, either by genetic manipulation of the progenitor cells or by isolating cells from patients (Freedman et al., 2015; Hale et al., 2018; Shamshirgaran et al., 2021). This opens the possibility of testing the reversal of genetic conditions using CRISPR-Cas9 technology (Forbes et al., 2018). Alternatively, the use of patient-derived organoids facilitates a true personalized medicine approach, including patient-specific drug validation (Low et al., 2019; van den Berg et al., 2019). Finally, external agents can be used to injure organoids, either to assess potential nephrotoxicity or to induce a renal disease phenotype. Employing omics techniques then allows investigation of new pathophysiological pathways for the identification of human translatable biomarkers (Harder et al., 2019). Some caveats do exist concerning the use of kidney organoids, such as their immaturity (although advances are being made (Garreta et al., 2019)), the presence of ‘non-renal’ cells (although strategies are evolving to circumvent this (Wu et al., 2018)), the lack of all cell types (due to their differentiation *via* a single pathway (Nishinakamura, 2019)), and the level of reproducibility among different iPSCs (Subramanian et al., 2019).

Advanced cell culture systems offer an opportunity to investigate disease mechanisms in a simpler manner than *in vivo*, yet provide some of the complexity of the kidney. Challenges remain in reconstituting the full gamut of renal physiology *in vitro* (Ashammakhi et al., 2018). Although throughput is necessarily much lower than 2D culture systems, capacities are ramping up with improved technologies such as the Draper system (Vedula et al., 2017; Fu et al., 2019). Differences seen in the responses to various stressors and drugs in these systems highlight the importance of adding layers of complexity to our toolbox. Further advances will come with the integration of multi-organ mimics to generate the so-called ‘human on a chip’ (Vernetti et al., 2017; Ramme et al., 2019). Adoption of such systems has been slow because of practical considerations (Ewart and Roth, 2020), but their potential for transforming translation from ‘bench to bedside’ cannot be over-emphasized. Furthermore, for ethical reasons the ability to forgo animal research would be priceless.

## 6 *Ex vivo* systems

*Ex vivo* approaches allow screening of compound efficacy and the assessment of target engagement and proof of mechanism, which inform the design of experiments *in vivo*. This is a crucial intermediate step between *in vitro* and *in vivo* work.

Isolated vascular and tubular structures can be used to understand underlying renal physiology and pathophysiology. The use of micro-dissected nephron segments (Helbert et al., 1997) and vascular segments has progressed from reverse transcription-polymerase chain reaction-based analyses determining changes in a few individual genes (Jensen et al., 2001), to large omics and functional studies using isolated perfused kidney tissue segments to gain a more refined physiological understanding (Giesecke et al., 2019). Expression data at the transcriptomic level (Lee et al., 2015) and more recently at the protein level (Limbutara et al., 2020) are a valuable resource that can facilitate investigations of segment-specific gene and protein expression patterns for novel targets. Isolated tubules and glomeruli have been used for mechanistic and functional studies, and experiments using perfused afferent and efferent arterioles have increased our understanding of the mechanisms regulating renal blood flow and GFR (Poulsen et al., 2011; Hansen, 2013; de Bruijn et al., 2015). The method is excellent for providing an in-depth understanding, although more difficult to use for high-throughput studies. Isolated renal vascular and microvascular segments *ex vivo* also afford investigation of renal endothelial and vascular smooth muscle cell function (Stulak et al., 2001) In addition, isolated rodent or human kidneys can be used to bridge from *in vitro* to *in vivo*, as well as confirming the translatability of a certain target (Taft, 2004; Weissenbacher et al., 2019).

### 6.1 Isolated glomeruli

Glomerular isolation *ex vivo* is driven by the need to study the glomerular compartment without tubular interference and the need to understand cell-cell and cell-matrix interactions. The advantage of this technique is the ability to isolate a complete glomerulus with all its original 3D structure, extracellular matrix, and complex renal cell composition. The drawback is that it is hard to recapitulate some important physiological features such as flow and pressure, thus limiting its relevance when studying glomerular endothelial function. However, there is additional benefit from isolating these structures directly from tissue compared with reconstituting them using cells and artificial devices, and include retaining the complex matrix structures that are important in the physiology of the overall structure. Isolated glomeruli are viable for up to 7–10 days for molecular analysis, pathway profiling, and omics characterization, and can be obtained from several sources: rodents, pigs, non-human primates, human biopsies, or human kidneys unsuitable for transplantation (Desideri et al., 2018; Rush et al., 2018; Wang et al., 2019). It is possible to apply both well-established molecular biology techniques and new approaches, such as high-content imaging and machine learning, to isolated glomeruli. Structures can be subjected to common stress factors such as cytotoxic chemical and various biological stressors, including cytokines, hypoxia, and ischemia-reperfusion, as well as mechanical/physical stressors (for example, flow and stretching), immune complexes, and immune cell co-culture methods. Isolated glomeruli can also be used for target identification, target validation, compound selection, phenotypic screening, proof-of-mechanism studies, molecular pathway characterization, and investigation of glomerular disease mechanisms.

### 6.2 Precision-cut kidney slices

Further to studying isolated glomeruli and tubules, precision-cut kidney slices (PCKS) allow the study of renal tissue in all its complexity while still in a defined culture environment, establishing a bridge between *in vitro* and *in vivo* studies (Stribos et al., 2016). Historically, PCKS have been used to study renal fibrosis, a very complex process that cannot be elucidated using *in vitro* systems. Two strategies can be used: slices can be taken from injured kidneys, for example, after unilateral ureteral obstruction (Genovese et al., 2016) or slices can be taken from healthy kidneys and treated with pro-fibrotic stimuli such as TGF-β (Stribos et al., 2017). An advantage of PCKS is that they can be cultured for 5–7 days, making them suitable for longitudinal studies and amenable to the types of analyses that are performed on kidneys isolated from mice. Histology and immunochemistry can be performed, and the kidney slices can be used for transcriptomic analyses (Bigaeva et al., 2019). It is worth noting that preparation of the kidneys for slicing induces stress, transiently increasing the expression of kidney injury markers, although these return to baseline after approximately 24 h. In addition to the well-established pro-fibrotic model, PCKS may be used to study the effect of inflammatory and hypoxic stimuli on kidney structure and injury.

Kidney slices can also be used as a predictive tool to test the efficacy of candidate drug compounds (Bigaeva et al., 2020) as part of initial screens before performing experiments *in vivo*. This can eliminate compounds are expected to show some efficacy in this model, but do not, and to determine an effect size of a compound and calculate group sizes for the experiments *in vivo*. The use of PCKS as a screening tool can reduce the number of animals needed to validate a test compound. As with brain slices, PCKS are also suitable for some imaging studies and can be superfused, for example, to examine tubule segment-specific changes in mitochondrial function (Stulak et al., 2001; Hall et al., 2009). PCKS represent a versatile tool to study aspects of kidney function and injury in an intact environment.

### 6.3 Zebrafish screen

Zebrafish are an efficient and high-throughput target validation system because they are genetically tractable and have a basic renal anatomy, with glomerular and tubular filtration processing (Wingert and Davidson, 2008). The pronephros in the zebrafish larvae consists of a single glomerulus connected to two tubular structures. The tubules express several of the important proximal and distal transporters, but lack the loop of Henle, because, as freshwater fish, they do not need to concentrate their urine. CRISPR-Cas9 is used to create insertions and deletions in the zebrafish genome to create loss-of-function models of targeted genes. Approximately 70% of human genes have at least one zebrafish ortholog, allowing validation of the majority of potential targets (Howe et al., 2017). The role of candidate genes in renal function is assessed by the proportion of larvae displaying edema and/or renal cysts, followed by detailed analysis of filtration and morphology (using electron microscopy and immunohistochemistry) (Hanke et al., 2015). This model will also capture the effects of altered gene expression in other organs, as well as any impact on embryogenesis, development, and survival (Gehrig et al., 2018). Zebrafish larvae may be used as an intermediate screening tool to triage the gene hit list, between cell culture/*ex vivo* systems and *in vivo* models in higher species, permitting quicker selection of potential targets. In addition, the adult zebrafish can be used as an efficacy model for studying both acute kidney injury and regeneration (McKee and Wingert, 2015) by using either nephrotoxic substances, laser ablation or genome editing to drive the injury (Morales and Wingert, 2017). Zebrafish are also commonly used as a safety model when screening for drug-induced kidney injury (Kato et al., 2020). However, cautious interpretation is warranted, as zebrafish physiology is far remote from human physiology.

## 7 Validating mechanism of action *in vivo*

**FIGURE 5:**
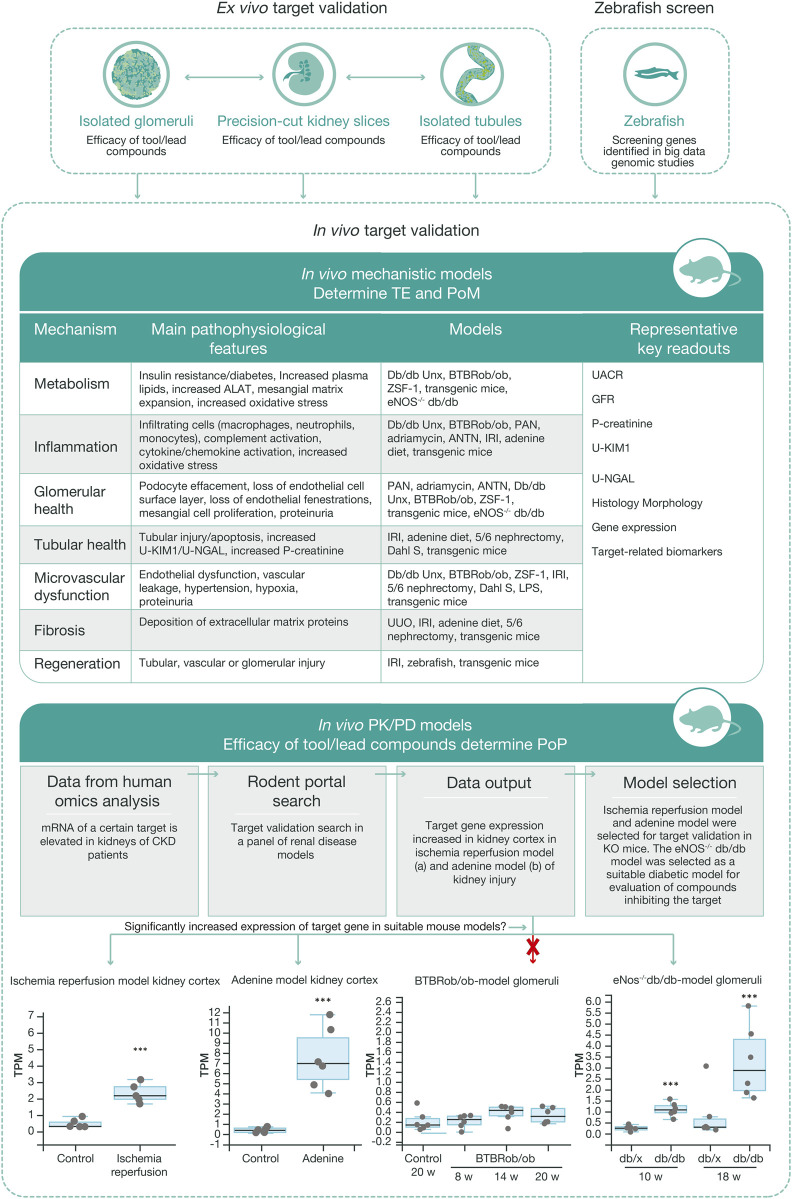
Workflow for *in vivo* target validation and compound testing. *Ex vivo* models are chosen to validate genes of interest and assess the efficacy of compounds targeting these gene products, based on their cellular expression and target engagement, respectively. Data from the *ex vivo* experiments guide the design of mechanistic and PKPD *in vivo* studies that generate data regarding target engagement and proof of mechanism, and on exposure and proof of principle, respectively. Various mechanistic models are used to validate targets and measure efficacy of test compounds.​ Selection of the most relevant *in vivo* PKPD model for a specific target or signaling pathway is based on *ex vivo* experimental data, human omics data, and RNA sequencing data from our ‘rodent portal’, generated from a panel of rodent renal disease models. The bottom panel illustrates differential upregulation of a target gene in different disease models, which in turn allows selection of the most appropriate model for the tested protocol. ALAT, alanine aminotransferase; ANTN, accelerated/non-accelerated nephrotoxic nephritis; BTBR, black and tan brachyuric; eNOS, endothelial nitric oxide synthase; IRI, ischemic reperfusion injury; KIM1, kidney injury molecule one; LPS, lipopolysaccharide; NGAL, neutrophil gelatinase; PAN, pyromycin aminonucleoside; PKPD, pharmacokinetic–pharmacodynamic; POM, proof of mechanism; TE, target engagement; UUO, unilateral ureter obstruction.

In parallel with data generation from *in vitro* and *ex vivo* models, the target validation process to define the most translatable *in vivo* model to provide proof of mechanism and proof of principle in CKD takes place. Similar to the workflows for *in vitro* and *ex vivo* models, several disease models with different mechanistic drivers may be used to capture the complexity of CKD (Figure 5). To facilitate selection of the most relevant preclinical model *in vivo* for a certain target, RNA sequencing can be performed on renal tissue from a panel of disease models to track the regulation of specific genes and signaling pathways. This information is also critical for an understanding the human translatability of the models in the context of disease and treatment mechanisms. The preclinical model is also selected based on the target biology, available biomarkers, and knowledge of the pathology of the disease models.

BOX 1 **Experimental design process for *in vitro* renal target validation**

1. Confirm that the target is the most effective and accessible target in the disease pathway2. Select a cell culture model in which the target is present3. Confirm a renal stressor that regulates the target in the same way as observed in patients with CKD more generally or a specific renal disease4. Confirm whether genetic modification (CRISPR knockout/overexpression) of the regulated target in the defined renal cell type results in a CKD-like phenotype5. Confirm that tool compounds or genetic modification can produce a treatment effect in a relevant translatable cell model6. Confirm whether genetic modification or tool compounds that agonize/antagonize a target in advanced models have an effect on clinical measures of renal function7. Continue target validation in advanced *in vitro*/*ex vivo* systems and establish a mode of action in translatable models *in vivo*

When designing a preclinical study, the aim is to use a mechanistic model in which target engagement can be easily captured, followed by a relevant proof-of-mechanism disease model in which an efficacy readout can be captured. The experiment in the mechanistic model is usually short and confirms that test molecule engages its target by measuring biomarkers directly linked to the target’s biology (activation of specific signaling pathways, altered gene or protein expression) in the plasma, urine, or in the kidney itself. During these studies, identification of additional markers of target engagement could influence the design of future clinical trials. For example, a viable target engagement model measuring the impact of a mineralocorticoid antagonist would measure plasma aldosterone and/or urinary electrolytes (Bamberg et al., 2018). This model is also used as a screening tool in the compound development process to ensure sufficient exposure in a subsequent pharmacokinetic–pharmacodynamic (PKPD) model. Once a compound (either an experimental molecule or a tool) has been shown to modify the disease pathway of interest at a relevant level of plasma or tissue exposure, a proof-of-mechanism study is designed. The model in this study should be relevant to the intended patient population and allow measurement of the physiological outcomes that will also be assessed in a clinical trial, such as urinary albumin excretion or a change in GFR, as potential human efficacy endpoint (Greene et al., 2019). Results from the proof-of-mechanism/PKPD model will affect the future design of clinical trials by guiding the modelling of the compound’s pharmacokinetics and calculation of receptor occupancy.

Translatability of models *in vivo* to human disease is a key aspect of target validation *in vivo* (Silver and Gerarduzzi, 2019). However, in most cases more than one disease model is needed to address mechanistic actions and functional readouts. A growing number of renal models *in vivo* have been developed (Betz and Conway, 2016; Mullins et al., 2016), representing damage to the different renal cell types that reflect key pathological changes seen in human disease. A good understanding of injury mechanisms in each model allows for the selection of a specific model to study a given target, based on its renal localization and mechanism of action. Therefore, selection of the most relevant disease model is crucial. For some candidate drugs the same model can address both the mechanism of action and functional efficacy; however, multiple models are usually necessary to build confidence. It is also important to recognize that any model of human kidney disease will capture only a ‘snapshot’ of the disease process (functional and/or structural), since human kidney disease occurs over decades.

Another approach to evaluating the relevance of a target is to use genetically modified animals for target validation and as safety tools to understand a super-physiological antagonization or agonization of gene function, as well as *in vivo* tool for ‘on/off’ target effects of candidate drugs. However, for several rodent preclinical models, inter-strain variability needs to be taken into account when selecting the disease model. For example, C57/Bl6 mice, one of the most commonly used background strains for generating knockout mice, are known to be less susceptible to renal injury than many other mouse strains (Breyer et al., 2005). In addition, issues like absence of human-like comorbidities or oftentimes impractical treatment regimen (e.g., preceding injury) may erode the predictive validity of a research model.

*In vivo* target validation together with the *in silico* and *in vitro* methodologies discussed earlier, is crucial to build a sound scientific understanding of the disease mechanisms in play.

## 8 Conclusion

CKD has steadily climbed the ranking of leading causes of death in recent years as a result of an aging population and increasing prevalence of risk factors such as diabetes and hypertension (Couser et al., 2011). Until now, research into finding therapies that be used to treat all forms CKD has focused on improving cardiovascular outcomes and in slowing disease progression, mainly with angiotensin-converting enzyme inhibitors and angiotensin receptor blockers, which also help to lower blood pressure, a driver of CKD progression, and more recent use of SGLT2 inhibitors. However, little progress has been made in halting or reversing disease progression.

A new patient-centric approach is key for future target identification and validation. Starting the target discovery based on patient data and a patient-centric approach using translatable models is essential to the identification and validation of targets that can result in successful CKD drug discovery programs and increase our understanding of mechanistic disease drivers. Scientific expertise in multiple areas of renal biology, pathophysiology and clinical aspects of renal disease is crucial, together with computational biology, systems-wide integration of omics with clinical data sets, and functional validation. Selection of the most appropriate *in vitro*, *ex vivo,* and *in vivo* systems for target validation and aiming to maximize translatability to patient disease can be guided by cutting-edge *in silico* and omics-based analyses.

The technological revolution continues to deliver patient omics data sets and AI systems for analysis of big data which allow researchers to use the patient as the starting point. Increased access to big data makes it impossible for one researcher to oversee all data. Therefore, the development of tools like knowledge graphs transform the way researchers work with big data. In recent years, much progress has also been made generating omics data from advanced *in vitro* models such as micro-physiological systems which facilitate studies of genes and pathways not possible in simple 2D cell culture systems. These advanced systems enable understanding of the complexity of the kidney by capturing the effect of 3D structure and cell-cell interactions. Despite the progress that has been made with advanced systems, there is still a need to capture adult renal cell biology, rather than the fetal signature that organoids present with today. In addition, more advanced models including multi-organ systems are needed to capture the full gamut of systemic responses and improve the validation tools for the future.

In parallel, the renal *in vivo* research community continue to build on the understanding of disease mechanisms and translatability by using metabolomics, proteomics, bulk and single cell NGS data in animal models. The omics data sets are key for understanding the translatability of animal models to patients. Owing to the complexity of chronic kidney disease with cellular crosstalk and multiorgan involvement, we still need to secure reliable *in vivo* models to support pharmacology read-out.

The increasing number of clinical trials for renal disease during the last decade have resulted in a better understanding of relevant endpoints, biomarkers and disease drivers. Clinical backtranslation beyond patient omics data will be a key factor and continue to improve therapeutic target identification and validation.

The toolbox of state-of-the-art models now available to us to simulate the complexity of CKD can facilitate obtaining readouts that are translatable from preclinical to clinical studies, which until now has been a major challenge for drug discovery in renal medicine. This toolbox needs to be continuously enriched by infusion of novel raw data acquired by researchers across the disciplines, as well as by development of powerful tools to mine and interpret those data. The recent years technological success will continue to deliver improved tools in the future. Despite the increased use of complex micro-physiological systems, there is still a need for testing *in vivo* for systemic pharmacological responses. We foresee that with the current progress in humanized *in vitro* models with increased complicity, the need for animal testing will decrease in the future. Using micro-physiological systems is expected to increase translatability to patients and reduce the number of animals used in pharmacological testing, a key ethical parameter in therapeutic target validation.

**FIGURE 6:**
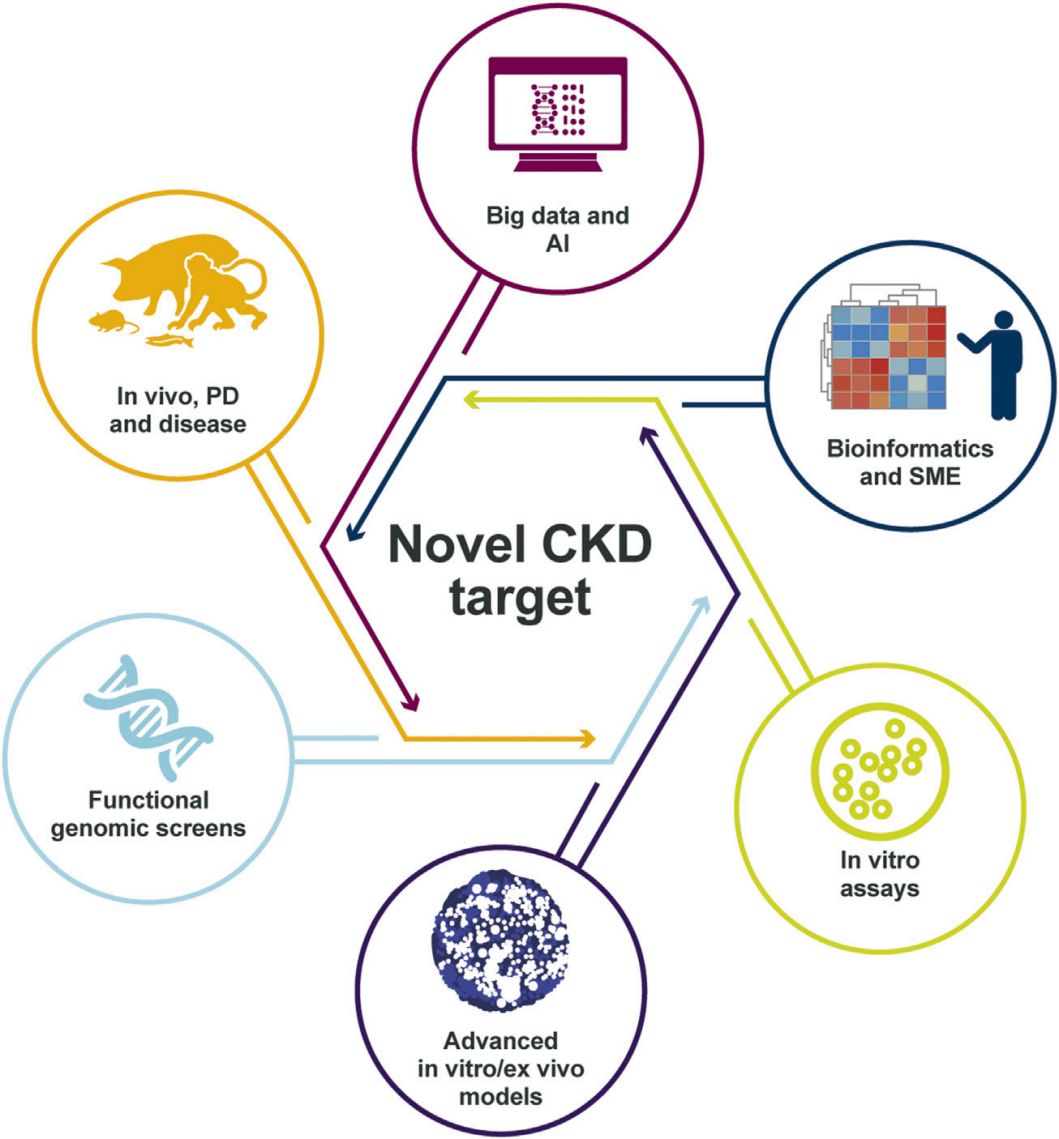
Identification of the right CKD target. The target identification and validation framework relies on multiple data sources and validation models, integrating many disease-relevant data sets, to create a holistic scientific understanding of the mechanisms that link the target to disease biology. The in-depth scientific understanding of pathophysiology and target link to disease is, in our view, essential for delivering successful medicines to patients with CKD in the future. AI, artificial intelligence; CKD, chronic kidney disease; PD, pharmacodynamics; SME, subject-matter expert.

In conclusion, the novel patient-centric approach building on the combination of *in silico* analysis of human data, together with extensive *in vitro* complex humanized models and *in vivo* validation in CKD research improves the probability to identify disease drivers that could be successful as potential drug targets (Figure 6).
